# Pure Autonomic Failure: A Case Report of Recurrent Orthostatic Hypotension

**DOI:** 10.31729/jnma.6480

**Published:** 2021-06-30

**Authors:** Prabin Khatri, Himal Panth, Sabina Khadka, Pramila Thapa, Rajshree Regmi, Sunil Shah, Sumit Gami, Ashutosh Upadhyaya, Mohammad Rizwan Alam, Srijana Sharma

**Affiliations:** 1 Department of Internal Medicine, Universal College of Medical Sciences, Bhairahawa, Nepal; 2 Universal College of Medical Sciences, Bhairahawa, Nepal; 3 Nepalese Army Institute of Health Sciences, Sanobharyang, Kathmandu, Nepal; 4 Lumbini Medical College, Palpa, Nepal

**Keywords:** *midodrine*, *orthostatic hypotension*, *pure autonomic failure*

## Abstract

Pure autonomic failure is a neurodegenerative disorder affecting the autonomic nervous system which clinically presents with orthostatic hypotension. It is a diagnosis of exclusion after detailed clinical examinations and relevant investigations. Here, we discuss a case of 68 years old male who had complaints of multiple episodes of loss of consciousness on standing from a sitting position for the last 3 years. The diagnosis was considered by clinical examinations revealing autonomic dysfunctions with normal appropriate investigations. The patient was treated successfully with midodrine, fludrocortisone, and other non-pharmacological interventions. We focused on doing various autonomic dysfunction tests in the evaluation of a patient with recurrent orthostatic hypotension. We suspect that pure autonomic failure might not have been considered in the differential diagnosis of recurrent orthostatic hypotension and suggest that it is to be kept as a differential in such a scenario. Midodrine has an effective role in syncope due to sympathetic vasoconstrictor failure.

## INTRODUCTION

Pure autonomic failure is a neurodegenerative disorder of the autonomic nervous system which usually presents with orthostatic hypotension.^1^ Meticulous history and detailed neurologic evaluation along with autonomic dysfunction tests will help rule out neurogenic causes of orthostatic hypotension. Orthostatic hypotension is more frequently associated with aging and secondary to diseases like diabetes, other autoimmune neuropathies, and multiple system atrophy and rarely due to pure autonomic failure. Disorders affecting the autonomic nervous system usually manifest with altered sweating, constipation, gastroparesis, impotence, syncope, and bladder dysfunction.^2^ We present a case of 68 years male presented with recurrent syncope and diagnosis of pure autonomic failure.

## CASE REPORT

A 68-year old male, from Gulmi, Nepal presented to Medicine OPD with complaints of multiple episodes of loss of consciousness on standing from a sitting position for the last 3 years. It was rapid in onset, short (usually <1 min) with spontaneous and complete recovery. He reported a history of dizziness and visual disturbance before the loss of consciousness. Dizziness was not related to the head position. He also complained of increased frequency of micturition, nocturia, constipation, and erectile dysfunction. There was no history of an unpleasant smell, fear, anxiety, abdominal discomfort before the loss of consciousness. On further questioning, he denied a history of aggravation by the meals, increase-ambient temperature, pain, the sight of blood, venipuncture, and intense emotion. There was a history of eyes opening during the loss of consciousness but no up rolling of eyes, frothing from the mouth, tongue bite, and fecal incontinence. On inquiry he didn't had history of drowsiness, headache, muscle pain, and disorientation after gaining consciousness. He denied a history of cough, chest pain, palpitation, breathlessness, vomiting, paralysis, and involuntary movement of any part of the body. He was a smoker for 15 years, but left 3 years back and was non-alcoholic. Family history was insignificant.

He visited many higher centers and was treated with fludrocortisone and advised to take plenty of fluids, but episodes were not relieved completely. On general examination, blood pressure fell from 130/100 mmHg in supine to 110/70 mmHg after standing for 3 minutes. Pulse rate was 70/min which did not increase on standing. His peripheral pulses were symmetrically palpable. There was no evidence of carotid bruits, pallor, icterus, lymphadenopathy, clubbing, cyanosis, edema, and dehydration. His systemic examination revealed no abnormal findings in the respiratory, cardiovascular, and gastrointestinal systems. Higher mental function, skull and spine, cranial nerves, motor and sensory, instance and gait examinations were unremarkable. For autonomic dysfunction, we did a skin examination which revealed no dryness and absence of hair, and pupillary examination was normal.

To rule out the possibility of secondary causes of orthostatic hypotension we investigated him with various routine and other possible tests including complete blood count and ESR, random blood sugar, renal function test, Thyroid function test, chest X-ray, HIV I and II, VDRL, EEG, ANA, RA factor and Extractable Nuclear Antigen panel; all of which appeared to be normal. ECG, Echocardiography, and Holter monitoring were done despite the absence of other cardinal symptoms of the cardiovascular system, which were within normal limits. The patient was undergone magnetic resonance imaging (MRI) head and electroencephalogram (EEG) despite having no progression in symptoms and no signs of central nervous system involvement, which came out to be normal. We thought of autonomic nervous system dysfunction in this case hence we proceeded for further clinical examination for the autonomic nervous system.

We did deep breaths test (pulse rate 90/min before and 82/min after the test), which showed a fall in pulse rate of <15 beats/min (normal subject shows fall of >15/ min). Handgrip test revealed diastolic pressure of 90 mmHg before and 100 mmHg after the test (normal subject shows a rise of diastolic pressure by >16 mmHg). The Valsalva maneuver showed; a ratio of the highest pulse rate in the preliminary rest period to the lowest pulse rate during the test to be <1.1 (normal subject shows >1.5). Carotid massage revealed a pulse rate of 92/min before and 88/min after the test. After getting the above positive findings on different tests for autonomic nervous system dysfunction and normal findings on other investigations to rule out a secondary cause of orthostatic hypotension; we made a diagnosis as a sympathetic vasoconstrictor deficiency (pure autonomic failure) with postural hypotension.

His symptoms were not improving despite taking fludrocortisone and adequate fluids from his previous visits at different centers. Hence, we decided to start his treatment with a vasopressor drug named midodrine along with fludrocortisone. We also advised him to take adequate fluids, sit with legs dangling over the bed edge before the first attempt to stand in the morning for several minutes and leg-crossing with maintained contraction of leg muscles for 30 seconds. On subsequent visits, there was a significant decrease in the event and showed that he has been taking the medication without side effects.

## DISCUSSION

Orthostatic hypotension is defined as a fall in systolic blood pressure of ≥20mmHg and/or diastolic blood pressure of ≥10mmHg within 3 minutes of standing or head-up tilt on a tilt table. It can be an initial presentation of autonomic dysfunction. In most cases, there is no compensatory increase in heart rate despite hypotension but for people with partial autonomic failure, heart rate may slightly increase which sometimes may not be enough to yield optimum cardiac output.^3^ It is one of the common causes of syncope. Transient loss of consciousness is a broader term that will not differentiate traumatic and nontraumatic causes and will include epileptic seizures and stroke.^4^

Syncope in general is of various types. Careful recognition of symptoms and appropriate investigations is critical in knowing the cause of syncope. Hence, we did thorough history taking, detail clinical examination and various investigations to rule out secondary causes of orthostatic hypotension. Cardiac syncope usually is sudden in onset without prodromal symptoms because perfusion to retina and cerebrum falls abruptly and there is no time to perceive and store information about warning signs (prodromal symptoms). In our case, there were no other cardinal cardiac symptoms besides syncope and a detailed cardiovascular system examination showed no abnormal clinical findings. Electrocardiogram, Echocardiography, and Holter monitor revealed normal results. Vasovagal syncope presents with warning signs in the younger group and rarely in older individuals probably because of retrograde amnesia in the older. Our patient denied a history of occurrence of syncope during or immediately after meals, due to increased ambient temperature, pain, the sight of blood, venipuncture, intense emotion, an episode during urination, cough, and defecation. Occipital ischemia may also present with pain in the neck and shoulders combined referred to as 'coat hanger pain'. Though the mechanism is unclear, sometimes autonomic dysfunction may also present with pain in certain postures in the lower back, buttock, and chest.^5^ Similar phenomenon was not present in our case. Syncope due to glossopharyngeal neuralgia presents with pain in the oropharynx, tonsils, or tongue before the loss of consciousness which was not present in our case.^2^

Diagnosis of autonomic dysfunction is missed in asymptomatic patients. If the aforementioned clinical clue is not present and diagnosis becomes inconclusive, an unmasking of asymptomatic symptoms is done by autonomic testing, especially in higher centers. Autonomic testing involves assessment of sympathetic cholinergic system by skin examination, pupillary examination, sympathetic adrenergic system by Valsalva maneuver and tilt-table test, the parasympathetic response by pulse rate variation in the deep breath test and Valsalva maneuver. These tests for autonomic dysfunction mainly help us to evaluate orthostatic hypotension from neural mediated syncope.^6^ In our case deep breaths test, handgrip test, Valsalva maneuver, and carotid massage revealed positive for autonomic dysfunction whereas skin examination and pupillary examination were normal. Because of unavailability, we could not perform the tilt-table test. Thus, there is a key role of autonomic nervous system tests in the early identification and treatment of either initially asymptomatic or symptomatic cases with diagnostic uncertainties.

Autonomic failure is caused by primary and secondary causes. Primary causes include degenerative neurologic diseases, like multiple-system atrophy, Parkinson's disease, Lewy body dementia, and pure autonomic failure, and secondary causes like autonomic peripheral neuropathies from diabetes, amyloidosis, Sjogren's syndrome, drug-induced, and HIV neuropathies.^7^ Autonomic failure due to multiple system atrophy presents with central involvement of sympathetic and parasympathetic systems and rapid progression of symptoms.^8^ Symptoms were non-progressive and with no central nervous system involvement in our case. Parkinson's disease and Lewy body dementia also present with motor symptoms in addition to autonomic involvement. Motor symptoms and dementia were not evident in our case. Our patient had an MRI of the head and EEG done at various centers which did not show abnormal findings. To rule out other secondary causes due to autonomic peripheral neuropathies, we did different laboratory investigations for diabetes, Sjogren's syndrome, syphilis, and HIV; which were normal.

On the above background, we made a suspicion of pure autonomic failure. Pure autonomic failure (PAF) typically presents in midlife or later with orthostatic hypotension or syncope. With extensive involvement, patients may present to a variety of specialties and may need multidisciplinary treatment approaches. Diagnosis is made by clinical signs of orthostatic hypotension without reactive tachycardia. Symptoms are compatible with chronic sympathetic deficiency without associated signs of central nervous system involvement. In patients with PAF, EEG and cerebral MRI are normal as in our case.^9^ Neurological examination of a patient with PAF will show no signs of movement disorders. Investigations and examinations to recognize an immunologic, deficiency, metabolic or toxic cause will be normal. It is diagnosed by exclusion of other disorders.^10^ The criteria given by the American Autonomic Society and the American Academy of Neurology in 1996 require that no other neurologic features should be present, it also highlights that some PAF may evolve into other synucleinopathies with central nervous system involvement.^1^ In our case, we found significant positive tests for autonomic dysfunction, no abnormal central nervous system examination results, and investigations underwent were normal. Thus, we concluded our diagnosis as sympathetic vasopressor deficiency (pure autonomic failure). If diagnosis and treatment are delayed, it may lead to seizures, pulmonary edema, retinal hemorrhage, and even renal insufficiency, myocardial infarction, cerebral hemorrhage, and ultimately death.^11^

Under treatment modalities, there are certain non-pharmacologic and pharmacologic measures. Non-pharmacological interventions include taking adequate fluids, sitting with legs dangling over bed edge before the first attempt to stand in the morning for several minutes, and leg-crossing with maintained contraction of leg muscles for 30 seconds.^2^ Pharmacologic interventions include fludrocortisone (mineralocorticoid) and midodrine (selective direct al-adrenergic agonist acting peripherally). Midodrine is the only Food and Drug Administration-approved drug for use in orthostatic hypotension.^12^ His symptoms were not decreasing despite having fludrocortisone and adequate fluids. Therefore, considering sympathetic vasoconstrictor failure, we started him on midodrine plus fludrocortisone along with advice on above mentioned non-pharmacological interventions, which significantly lowered his frequency of syncope. On subsequent visits, there was a significant decrease in the event and showed that he has been taking the medication without side effects.

We focused on performing various autonomic dysfunction tests in the evaluation of a patient with recurrent orthostatic hypotension. Diagnosis of pure autonomic failure might be missed in the case of recurrent orthostatic hypotension, hence we suggest it to be kept as the differential diagnosis in such a condition. Not only fludrocortisone, adequate fluids, and non-pharmacological measures, midodrine also has an effective role in syncope due to sympathetic vasoconstrictor failure.
